# High Levels of Cerebrospinal Fluid Kappa Free Light Chains Relate to IgM Intrathecal Synthesis and Might Have Prognostic Implications in Relapsing Multiple Sclerosis

**DOI:** 10.3389/fimmu.2022.827738

**Published:** 2022-03-07

**Authors:** Jéssica Castillo-Villalba, Sara Gil-Perotín, Raquel Gasque-Rubio, Laura Cubas-Nuñez, Sara Carratalà-Boscà, Carmen Alcalá, Carlos Quintanilla-Bordás, Francisco Pérez-Miralles, Cristina Ferrer, Antonio Cañada Martínez, Jordi Tortosa, Luís Solís-Tarazona, Luisa Campos, Alberto Leivas, Begoña Laíz Marro, Bonaventura Casanova

**Affiliations:** ^1^Neuroimmunology Unit, Neurology Department and Health Research Institute, Hospital Universitario y Politécnico La Fe, Valencia, Spain; ^2^Data Science, Biostatistics and Bioinformatics, Health Research Institute, Hospital Universitario y Politécnico La Fe, Valencia, Spain; ^3^Clinical Laboratory, Hospital Universitario y Politécnico La Fe, Valencia, Spain; ^4^Neurology Department, Hospital Dr. Peset, Valencia, Spain; ^5^Scientific Department, The Binding Site Iberia, Barcelona, Spain

**Keywords:** ectopic lymphoid follicles, CHI3L1, high-efficacy disease-modifying therapies, prognostic factor, oligoclonal band

## Abstract

Cerebrospinal kappa free light chain (KFLC)-index is a marker of intrathecal immunoglobulin synthesis that aids in the diagnosis of multiple sclerosis (MS). However, little evidence exists on its prognostic role. Our aim is to analyze the relationship between KFLC-index and other MS biomarkers and to explore its prognostic role. This is a monocentric observational study in a cohort of 52 people with relapsing MS (pwRMS) performed on prospectively acquired clinical data and with retrospective evaluation of biomarkers. We measured KFLC-index, immunoglobulin intrathecal synthesis, cerebrospinal fluid (CSF) chitinase 3-like 1 (CHI3L1), and neurofilament light protein (NFL) and reviewed MRI to detect leptomeningeal contrast enhancement (LMCE). We compared time to Expanded Disability Status Scale (EDSS) 3 and to initiation of high-efficacy disease-modifying therapies (heDMTs) by multivariate Cox regression analysis. Median KFLC-index correlated with IgG/IgM indexes (*p* < 0.0001/*p* < 0.05) and IgG-oligoclonal bands (OCGBs) (*p* < 0.001). Patients with IgM-oligoclonal bands (OCMBs) had a higher KFLC-index (*p* = 0.049). KFLC-index was higher in patients with LMCE (*p* = 0.008) and correlated with CHI3L1 (*p* = 0.007), but disease activity had no effect on its value. Bivariate and multivariate analyses confirmed KFLC-index > 58 as an independent risk factor for reaching an EDSS of 3 (hazard ratio (HR) = 12.4; 95% CI = 1.1–147; *p* = 0.047) and for the need of treatment with heDMTs (HR = 3.0; 95% CI = 1.2–7.1; *p* = 0.0013). To conclude, our data suggest a potential prognostic role of the KFLC-index during the MS course.

## Background

Multiple sclerosis (MS) has long been regarded as a T cell-mediated disease. However, increasing evidence supports the pathogenic role of B cells, through both the production of antibodies and the release of proinflammatory factors in the cerebrospinal fluid (CSF) (1). Antigen-directed affinity maturation and terminal differentiation of B cells contributing to immunoglobulin synthesis occur within perivascular infiltrates and meningeal ectopic lymphoid-like follicles and have been related to gray matter pathology and progressive disease (2–4).

Due to a predictive role in clinically isolated syndrome (CIS) conversion to MS, the examination of cerebrospinal IgG-oligoclonal bands (OCGBs) has been reintroduced in the latest MS diagnostic criteria (5). This paradigm change has focused attention on other biomarkers related to immunoglobulin syntheses, such as kappa free light chains (KFLCs) and IgM-oligoclonal bands (OCMBs). KFLC, a secreted remnant produced by plasma cells, whose values can be corrected for the blood–brain barrier permeability by use of the KFLC-index, has been described as a reliable marker of intrathecal IgG synthesis in MS (6–9), but there are scarce prognostic data on excess of KFLC in the long term. Alternatively, interest in OCMBs, present in 48% of people with MS (pwMS), has been recently renewed after increased research supporting a correlation with unfavorable disease courses in MS (10–13). Considering this background, the present single-center observational study in relapsing MS (RMS) patients aims to assess the potential relationship between KFLC-index and other neuroinflammatory biomarkers, such as OCMBs, and its value as a complementary tool to OCGBs and as a risk factor for neurological disability and treatment failure.

## Materials and Methods

### Participants

This is a single-center observational study in a cohort of 52 pwMS with a first demyelinating episode and definite diagnosis of RMS according to the 2017 McDonald criteria (5). A cohort of consecutive and unselected patients was recruited in the period comprised between 2010 and 2019 based on the availability of CSF at the time of disease onset. Clinical data were acquired prospectively, and biomarker and radiological data were analyzed retrospectively. Disability was estimated according to the Expanded Disability Status Scale (EDSS) at the time of lumbar puncture (LP) and in three-monthly visits during follow-up. The clinical study was approved by the Institutional Ethics Committee (reference number PI17/01544).

### Definitions

A clinical attack or demyelinating event (relapse) was defined as an acute worsening of neurologic function lasting more than 24 h, not explained by fever or physical stress, and followed by a variable degree of recovery. MRI assessment of acute demyelination was required. CSF samples were contemporary to disease onset.

RMS patients were treated with disease-modifying therapies (DMTs) according to routine clinical practice. In general, a first-line DMT was offered unless any of the following circumstances occurred: i) two clinical attacks in a year, ii) a clinical attack and/or a new gadolinium-enhanced lesion (GEL), or iii) a disabling clinical attack with residual EDSS of at least 2 points in the pyramidal system. In these cases, and in those with treatment failure, high-efficacy DMTs (heDMTs) were preferred. We considered as heDMTs cladribine, fingolimod, natalizumab, ocrelizumab, rituximab, alemtuzumab, and hematopoietic stem cell transplantation (aHSCT). ﻿Treatment failure was considered in case of clinical relapse followed by MRI demonstrating GEL within 3 months from the flare.

### Laboratory Analysis in the Cerebrospinal Fluid

Paired CSF and serum samples were collected simultaneously and stored at −80°C. Serum and CSF were tested to rule out infections, monoclonal serum OCB, or other inflammatory systemic diseases. IgG, IgM, and albumin were measured in CSF and serum with immuno-nephelometry, and Link index was calculated to obtain IgG and IgM indexes to correct the influence of blood–barrier permeability in the immunoglobulin concentration as per the following formula:

*Ig index: (CSF Ig/serum Ig)/(CSF albumin/Serum albumin)*

Detection of oligoclonal IgG and IgM bands was performed by isoelectric focusing and immunoblotting, as previously described (14). KFLC was measured by turbidimetry using the Freelite Mx immunoassay (The Binding Site, Birmingham, UK) on the automated analyzer Optilite (The Binding Site). Serum samples were diluted 1/10, and CSF samples were analyzed undiluted as recommended by the manufacturer. The lower limit of quantification (LOQ) for KFLC in the CSF was 0.03 mg/L. KFLC-index was calculated with reference to albumin CSF and serum albumin according to the following formula:

*KFLC-index: (CSF KFLC/serum KFLC)/(CSF albumin/Serum albumin)*

CSF NFL and chitinase 3-like 1 (CHI3L1) levels were assessed by ELISA using commercially available kits according to the manufacturer’s instructions (Uman Diagnostics AB, Umea, Sweden; and Quantikine ELISA kit, R&D Systems, Minneapolis, MN, USA, respectively). Samples were run in duplicate in a blind manner. The intra-assay variability was 5.8% for NFL and 5.13% for CHI3L1. The mean inter-assay coefficients of variation were 4.5% and 5.2%, respectively.

### MRI Detection of Post-Gadolinium Leptomeningeal Enhancement

After CSF collection, 3-Tesla MRI acquisitions in all patients were analyzed. Sequences included pre-contrast T2-fluid-attenuated inversion recovery (T2-FLAIR) and post-contrast three-dimensional (3D) T2-FLAIR scans (1 × 1 mm) performed at least 10 min after IV injection of a single dose of gadolinium (Gd) (0.1 mmol/kg) and 3D T1 sequences (General Electric, Signa HDx, Boston, MA, USA) according to published scan acquisition parameters (15). Pre-contrast T2-FLAIR scans were available for all patients. Leptomeningeal contrast enhancement (LMCE) was defined as signal intensity within the subarachnoid space that was substantially greater than that of brain parenchyma on post-contrast 3D FLAIR images, excluding high-signal regions adjacent to dural venous sinuses, basal meninges, and large subarachnoid veins (15). For this manuscript, two trained raters (SG-P and SC-B) were involved in analyzing the images, and inter-rater variability was <1%.

### Statistical Analysis

Continuous variables were reported as median with interquartile range (IQR) and categorical variables as absolute frequencies and percentages. Data did not distribute normally and were analyzed with non-parametric tests. Groups were compared with the Wilcoxon rank sum and Kruskal–Wallis tests, and correlations were performed with Spearman’s test. All associations were adjusted for sex, age, and disease duration. Bonferroni post-hoc adjustment was performed in the case of multiple comparisons. A two-sided p-value of less than 0.05 was considered to indicate statistically significant differences. ﻿ Time to disability endpoints (EDSS score of 3) and initiation of heDMTs were compared using the Kaplan–Meier curves and a log-rank test. Patients who did not reach EDSS scores of >3 or who maintained first-line DMTs during follow-up were considered censored at the time of the last clinical assessment. We performed Cox proportional hazard regressions to estimate the hazard ratios (HRs) along with 95% CI as measures of association between test results and endpoints. Adjustments were made for potential confounding factors (sex, age, and EDSS at LP). Statistical analyses were performed using R studio 2021.09 (https://www.R-project.org/).

## Results

### Participant Characteristics

﻿Fifty-two people with RMS (pwRMS) were enrolled in this study. Forty-two patients (81%) were women, and the median age at onset was 35 (25–45) years. Patients were followed up for a median of 58 (45–75) months. The median baseline disability score (EDSS) was 2.0 (1–2). Forty-two patients (81%) were experiencing clinical relapse, and 26 (50%) had GEL in the MRI at the time of CSF collection. Two patients were receiving first-line DMTs at the time of LP, one patient had received corticosteroids, and one had been treated with cyclophosphamide in the previous 2 months. Demographic and clinical data of all patients are summarized in **Table 1**.

**Table 1:** Demographic, clinical, and MRI data of pwMS in the study.

N	52
Age	35.5, 15–71
Female	42 (81)
OCGBs	42 (81)
OCMBs	29 (55)
EDSS at LP	2, 1–2
EDSS at FU	2, 1–3
Duration follow-up (years)	58 (45–75)
Clinical attack	42 (81)
GEL	26 (50)
Presence of LCME	13 (25)
DMT at LP	3 (5.7); 1 (corticosteroids*)
Kappa index	47.8, 14.3–130

Data are shown in the following order: quantitative variables: median, p25–p75, and categorical variables: number (percentage).

OCB, oligoclonal bands G and M; EDSS, Expanded Disability Status Scale; LP, lumbar puncture; FU, last follow-up; GEL, gadolinium-enhanced lesions; DMT, disease-modifying therapy; heDMT, high-efficacy DMT; pwMS, people with multiple sclerosis.

^*^Less than 3 months from LP.

### Kappa Free Light Chain-Index Association With Diagnostic and Prognostic Multiple Sclerosis Biomarkers

**Figure 1:**
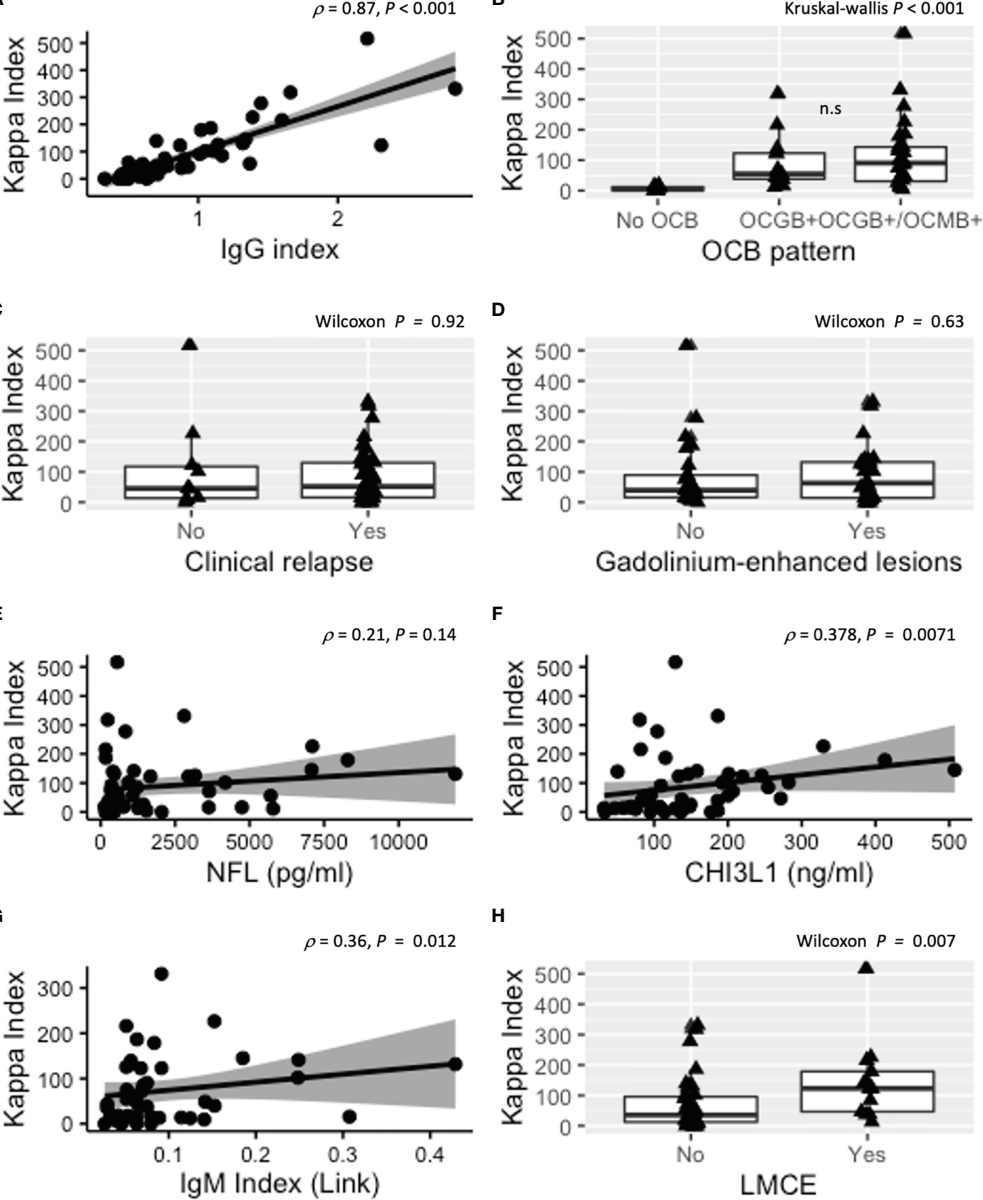
Association of KFLC-index with other diagnostic and prognostic MS biomarkers. **(A)** Correlation between KFLC-index and IgG index. **(B)** KFLC-index was significantly increased in the presence of OCGBs with respect to patients without intrathecal immunoglobulin synthesis. KFLC-index tended to be higher in the presence of both OCGBs and OCMBs but did not correlate with concurrence of clinical relapse **(C)** or presence of GEL **(D)**. **(E)** KFLC-index did not correlate with CSF NFL but did weakly with CSF CHI3L1 and IgM index [**(F, G)**, respectively]. **(H)** Patients with LMCE had higher median values of KFLC-index. Kappa index, kappa free light chain (KFLC) index; OCB, oligoclonal band; GEL, gadolinium-enhanced lesions; NFL, neurofilament light chain protein; CSF, cerebrospinal fluid; CHI3L1, chitinase 3-like 1; LMCE, leptomeningeal contrast enhancement; MS, multiple sclerosis. n.s., not significant.

Median KFLC-index was 47.8 (14.3–130) and was significantly higher in pwMS with OCGBs as compared to those without (4.63 (0.03–11.76) and 73.14 (35.72–137.5); *p* < 0.0001, respectively). Likewise, the correlation with IgG index was strong (ρ = 0.872; *p* < 0.0001) (**Figures 1A, B**). In the receiver operating characteristic (ROC) analysis, a KFLC-index cutoff value of 12.8 predicted the presence of OCGBs with a sensitivity of 93% and a specificity of 80%, while a cutoff value of 17.2 was more specific (sensitivity 83% and specificity 90%). The area under the curve (AUC) was 0.952. A contingency analysis for both cutoff values showed a positive predictive value (PPV) of 94.7%/82% and a negative predictive value (NPV) of 80%/90%.

During active disease, defined as relapse or presence of GEL in the MRI simultaneous to CSF collection, KFLC-index in our cohort was not significantly different from that in pwMS on remission (*p* = 0.92 and *p* = 0.63, respectively) (**Figures 1C, D**). Likewise, KFLC-index was not associated with sex (*p* = 0.76), age (*p* = 0.93), EDSS (*p* = 0.11), or DMTs at time of CSF collection (Kruskal–Wallis *p* = 0.59 with no differences between non-treated and treated with first-line DMTs or second-line DMTs). When we analyzed other cerebrospinal biomarkers such as neurofilament light protein (NFL) and chitinase 3-like 1 (CHI3L1), we only found a weak correlation between KFLC-index and CHI3L1 (ρ = 0.378; *p* = 0.007) (**Figures 1E, F**).

Twenty-nine patients (55.7%) had simultaneous OCGBs and OCMBs. KFLC-index was higher in pwMS with OCMBs (85.3 (17.9–141.4)) than in those without (37.8 (12.3–65.3); *p* = 0.049). The correlation between KFLC and IgM index was significant but less robust than with OCGBs (ρ = 0.36; *p* < 0.012) (**Figure 1G**). We sought for the discriminative potential of KFLC-index to detect both double-positive (OCGBs and OCMBs) patients, and we observed that pwMS with unique OCGB pattern had a lower median KFLC-index (54.9 (38.4–122.8)) as compared to those with OCGBs and OCMBs (90.6 (30.7–143.3)), but this difference did not reach statistical significance (*p* = 0.635) (**Figure 1B**).

Finally, as ectopic leptomeningeal follicles (eLFs) are thought to be involved in the intrathecal production of immunoglobulins, we studied the potential association between KFLC-index and LMCE, the radiological counterpart of eLFs. The presence of LMCE was observed in 13 patients (25%). Patients with LMCE had higher KFLC-index than patients without LMCE (123 (46.8–178.8) and (35.0 (12.4–96), respectively) (*p* = 0.008) (**Figure 1H**). There was a patient with LMCE and an outlier KFLC-index value of 516. After withdrawal from the analysis, the KFLC-index association with LMCE was still significant (*p* = 0.019).

### Kappa Free Light Chain-Index as Prognostic Factor of Disability and Initiation of High-Efficacy Disease-Modifying Therapy

**Figure 2:**
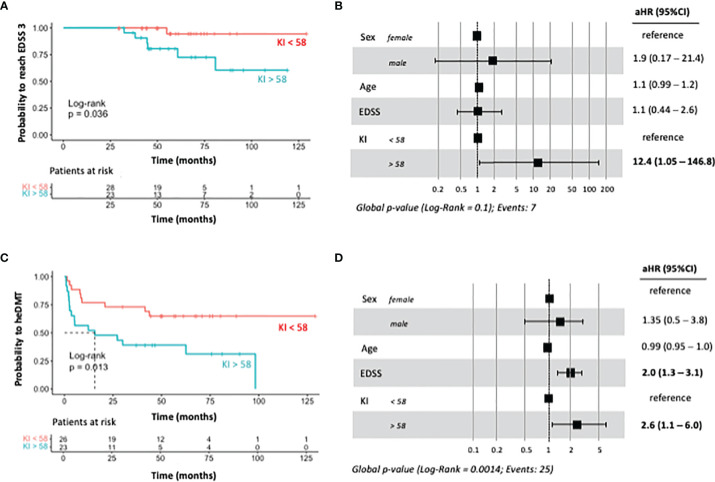
Bivariate and multivariate regression models for predicting the risk of reaching EDSS score of 3 and of treatment escalation. **(A)** Kaplan–Meier survival analysis showing the probability for reaching an EDSS of 3 stratified by KFLC-index value of 58. **(B)** Forest plot of multivariate analysis for the same disability endpoint adjusted for sex, baseline age, and baseline EDSS. **(C)** Kaplan–Meier survival analysis showing the probability for treatment escalation to heDMT stratified by KFLC-index value of 58. **(D)** Forest plot of multivariate analysis for the same disability endpoint adjusted for sex, baseline age, and baseline EDSS. KFLC-index, kappa free light chain index; EDSS, expanded disability severity score; heDMT, high-efficacy disease-modifying therapy.

To explore time to disability endpoint (EDSS score of 3) and time to initiation of heDMTs, patients were stratified according to the most discriminative KFLC-index cutoff value. Seven patients (13.4%) had reached an EDSS score of 3 at the last data update. In the bivariate analysis, KFLC > 58 was a risk factor to reach EDSS of 3 (log-rank *p* = 0.036) (**Figure 2A**). Cox multivariate regression analysis (adjusted for sex and baseline age and EDSS) confirmed that KFLC-index > 58 was an independent risk factor for this outcome (HR) = 12.4, 95% CI = 1.1–147 and *p* = 0.047 (**Figure 2B**).

The need for initiation of heDMTs during follow-up occurred in 25 patients (48%). The Kaplan–Meier survival analysis showed that pwMS with KFLC > 58 required heDMTs with a median time of 18 months (log-rank *p* = 0.013) and was an independent risk factor in the multivariate analysis (HR = 2.6; 95% CI = 1.1–6.0 p = 0.0014) (**Figures 2C, D**). Baseline EDSS was also an independent risk factor for treatment with heDMTs (HR 2.0, 95% CI 1.3– 3.1; *p* = 0.01).

## Discussion

KFLC in the CSF is a product of immunoglobulin intrathecal synthesis and is measured with a quantitative and automated method, not dependent on technical interpretation. Here we showed that KFLC^CSF/serum^ index is a feasible alternative to OCGBs and/or IgG index for MS diagnosis that is not significantly affected by concurrent radiological or clinical disease activity. Regarding long-term prognosis, KFLC-index correlated with CHI3L1, and its excess was related to the presence of OCMBs and LMCE, biomarkers associated with worse outcomes, progressive disease, and gray matter pathology. Additionally, KFLC-index above 58 was an independent risk factor for reaching an EDSS score of 3 and predicted the need for high-efficacy treatments during follow-up.

KFLC-index has been shown to be a sensitive marker of intrathecal immunoglobulin synthesis comparable to OCGB detection, the current “gold standard” (16). However, non-consensus has been reached about the most informative threshold value. **Supplementary Table 1** summarizes the distinct studies addressing KFLC cutoff values. In our study, all patients fulfilled the criteria for RMS; thus, we performed ROC analysis in comparison with OCGBs, achieving a good sensitivity/specificity relation with a KFLC-index cutoff of 12.8. The specificity increased with a higher cutoff value, as may also be inferred by other groups’ reported results on CIS/MS patients. The discrepancies between different thresholds could be due to variability between the clinical cohorts included in the evaluation and/or different measurement techniques, although the best-performing cutoff value might depend on the specific diagnostic context.

In our cohort, KFLC-index was not related to inflammatory relapse or GEL, suggesting that there might not be a relationship between KFLC production and acute inflammatory activity in MS. These findings are in agreement with previous studies that showed no correlation between KFLC-index in pwRMS patients with GEL on MRI at the time of sampling (17, 18). However, another work described a significantly higher KFLC-index in patients presenting with myelitis or with >9 T2 lesions in the MRI (19).

The long-term prognostic role of KFLC is as yet widely unexplored. We showed that KFLC median values increased in the presence of OCMBs and correlated with the IgM index. KFLC might have the potential to detect IgM-producing cell clones commonly found in early inflammatory responses (20), similarly to what occurs during IgG synthesis, as has been suggested by Süße et al. (21). IgM intrathecal synthesis is associated with a shorter time between relapses and predicts earlier conversion to secondary progressive MS and higher neurological disability measured by EDSS (11, 13, 22). It also correlates with greater T2 lesion load and atrophy (23). Thus, our findings suggest that KFLC-index could play a potential role as an indirect indicator of intrathecal IgM synthesis that might predict worse outcomes in pwMS. CHI3L1 is a protein related to progressive disease and long-term disability in MS (24). Comabella et al. showed a correlation between OCMBs and CHI3L1 (25), and because our results suggest that KFLC-index is related to OCMBs, it might support a potential correlation with CHI3L1. Indeed, we found a weak correlation between KFLC-index and CHI3L1 cerebrospinal levels, but other studies have not confirmed this finding (17). Meningeal eLFs are reported to be the origin of intrathecal immunoglobulin production (2), although, to our knowledge, a potential link between eLFs and KFLC-index has not been described yet. In our cohort, the presence of at least one LMCE in the MRI was significantly associated with a higher KFLC-index. eLFs contribute to cortical demyelination and progression in MS (26), and therefore excess KFLC may also be a predictor of persistent cortical inflammation. This hypothesis strengthens the idea of an association between humoral and innate immune-inflammatory processes involved in the progression of disability.

While KFLC-index did not correlate to baseline EDSS, bivariate and multivariate analyses showed that a KFLC-index over 58 was a risk factor for reaching a disability score of 3 (EDSS) and increased the likelihood of treatment initiation with heDMTs. Similarly, an excess of KFLC-index has been associated with a higher probability of CIS conversion to MS (9), and patients with KFLC-index >100 at baseline had twice as high a probability of a second clinical attack within 12 months (27). Patients with higher baseline values of KFLC achieved EDSS-confirmed progression in shorter periods and showed a significant positive correlation with disability at follow-up, independently of the effects of age, OCB status at baseline, and DMT exposure (18, 28–30). Our conclusions should be interpreted cautiously due to study limitations such as low sample size, the low incidence in prognostic endpoints, and higher incidence of OCMBs as compared to other studies that might have introduced potential sources of bias. Also, the kinetics of CSF KFLC are not completely known and could be influenced by renal function, not investigated in this study.

## Conclusions

Overall, this work indicates a potential link between KFLC, OCMBs, and eLFs and suggests an added prognostic value for KFLC measurement. This, added to a feasible implementation of the technique in most clinical laboratories, should prompt the performance of larger prospective studies.

## Data Availability Statement

The raw data supporting the conclusions of this article will be made available by the authors, without undue reservation.

## Ethics Statement

The studies involving human participants were reviewed and approved by the local (Hospital La Fe) Institutional Ethics Committee (reference number PI17/01544). The patients/participants provided their written informed consent to participate in this study.

## Author Contributions

BC is the head of the neuroimmunology laboratory. SG-P and BC designed the study, attended the patients, gathered and organized the clinical data, analyzed the data, wrote the manuscript, and revised the final draft. JC-V performed the biomarker analysis and wrote the manuscript. LC-N, RG-R, LS-T, and SC-B helped in gathering the biomarker and image data and revised the manuscript. FP-M, CA, and CQ-B attended the patients and helped in the registration of the clinical data. BM and JT helped to collect the analytical data and revised the manuscript. AM performed the statistical analysis. LC and AL revised the manuscript and gave statistical assistance. CF has been helping out in attending patients and in gathering of data. All authors listed have made a substantial, direct, and intellectual contribution to the work and approved it for publication.

## Funding

This study has been funded by the Health Research Institute Carlos III (ISCIII), Ministry of Health (PI17/01544; PI020/01446), and FEDER funding. SG-P is financed by Juan Rodes Clinical Research Fellowship (ISCIII-JR20/00033).

## Conflict of Interest

Authors LC and AL were employed by The Binding Site Iberia. The Binding Site has ceded the turbidimetry kits to measure KFLC for the present study.

The authors declare that the research was conducted in the absence of any commercial or financial relationships that could be construed as a potential conflict of interest.

## Publisher’s Note

All claims expressed in this article are solely those of the authors and do not necessarily represent those of their affiliated organizations, or those of the publisher, the editors and the reviewers. Any product that may be evaluated in this article, or claim that may be made by its manufacturer, is not guaranteed or endorsed by the publisher.

## Abbreviations

CHI3L1, chitinase 3-like 1; CIS, clinically isolated syndrome; CSF, cerebrospinal fluid; DMT, disease-modifying therapy; EDSS, Expanded Disability Status Scale; eLFs, ectopic leptomeningeal follicles; GEL, gadolinium-enhanced lesion; heDMT, high-efficacy disease-modifying therapy; KFLC, kappa free light chain; LMCE, leptomeningeal contrast enhancement; LP, lumbar puncture; MS, multiple sclerosis; NFL, neurofilament light protein; OCGBs, IgG-oligoclonal bands; OCMBs, IgM-oligoclonal bands; pwRMS, people with relapsing MS.
