# Supplementary material for: High Levels of Cerebrospinal Fluid Kappa Free Light Chains Relate to IgM Intrathecal Synthesis and Might Have Prognostic Implications in Relapsing Multiple Sclerosis
Source: Front Immunol. 2022 Mar 7;13:827738. doi: 10.3389/fimmu.2022.827738 (PMC8940299; doi:10.3389/fimmu.2022.827738)
Supplement: Supplementary file 1 [file Table_1.docx]

**Supplementary table 1. KFLC index cut-off values for CIS/MS diagnosis**

| **Cut-off** | **Sensitivity** | **Specificity** | **Measurement** | **Type of patient (N)** | **Reference** |
| --- | --- | --- | --- | --- | --- |
| 20 | 70 | 82 | Nephelometry | MS (33), “possible MS” (15), C (41) | Desplat-Jégo et al., 2005(1) |
| 12 | 91 | 95 | Nephelometry | NIND (33), non-MS IND (24), MS (23) | Duranti et al.,2013 (2) |
| 12.3 | 93 | 100 | Nephelometry | NIND (73), non-MS IND (33), MS (71) | Pieri et al., 2013(3) |
| 5.9 | 96 | 86 | Nephelometry | CIS (60), MS (60), NIND (60), C (420) | Presslauer et al, 2016(4) |
| 10.5 | 87 | 76 | Nephelometry | NIND (179), IND (115) | Gurtner et al., 2018(5) |
| 10.7 | 93 | 96 | Turbidometry | NIND/IND (70), RRMS (29) | Menendez-Valladares et al., 2015(6) |
| 6.6 | 93 | 83 | Turbidometry | MS (284), C (219) | Leurs et al., 2020(7) |
| 7.8 | 89 | 83 | Nephelometry | MS (64), NIND (82) | Gaetani et al., 2020(8) |
| 10.6 | 86 | 89 | Nephelometry | MS (64), IND (24) | Gaetani et al., 2020(8) |
| 3.0 | 98 | 86 | Turbidometry | MS (45), NIND/IND (207) | Sanz-Díaz et al., 2021(9) |

MS: multiple sclerosis; RRMS: relapsing-remitting MS; CIS: clinically-isolated syndrome; NIND: non-inflammatory neurological disease; IND: inflammatory neurological disease; C: control.

1. Desplat-Jégo S, Feuillet L, Pelletier J, Bernard D, Chérif AA, Boucraut J. Quantification of immunoglobulin free light chains in cerebrospinal fluid by nephelometry. *J Clin Immunol* (2005) doi:10.1007/s10875-005-5371-9

2. Duranti F, Pieri M, Centonze D, Buttari F, Bernardini S, Dessi M. Determination of kFLC and K index in cerebrospinal fluid: A valid alternative to assessintrathecal immunoglobulin synthesis. *J Neuroimmunol* (2013) doi:10.1016/j.jneuroim.2013.07.006

3. Pieri M, Storto M, Pignalosa S, Zenobi R, Buttari F, Bernardini S, Centonze D, Dessi M. KFLC Index utility in multiple sclerosis diagnosis: Further confirmation. *J Neuroimmunol* (2017) doi:10.1016/j.jneuroim.2017.05.007

4. Presslauer S, Milosavljevic D, Huebl W, Aboulenein-Djamshidian F, Krugluger W, Deisenhammer F, Senel M, Tumani H, Hegen H. Validation of kappa free light chains as a diagnostic biomarker in multiple sclerosis and clinically isolated syndrome: A multicenter study. *Mult Scler* (2016) doi:10.1177/1352458515594044

5. Gurtner KM, Shosha E, Bryant SC, Andreguetto BD, Murray DL, Pittock SJ, Willrich MA V. CSF free light chain identification of demyelinating disease: Comparison with oligoclonal banding and other CSF indexes. *Clin Chem Lab Med* (2018) doi:10.1515/cclm-2017-0901

6. Menéndez-Valladares P, García-Sánchez MI, Cuadri Benítez P, Lucas M, Adorna Martínez M, Carranco Galán V, García De Veas Silva JL, Bermudo Guitarte C, Izquierdo Ayuso G. Free kappa light chains in cerebrospinal fluid as a biomarker to assess risk conversion to multiple sclerosis. *Mult Scler J - Exp Transl Clin* (2015) doi:10.1177/2055217315620935

7. Leurs CE, Twaalfhoven HAM, Lissenberg-Witte BI, van Pesch V, Dujmovic I, Drulovic J, Castellazzi M, Bellini T, Pugliatti M, Kuhle J, et al. Kappa free light chains is a valid tool in the diagnostics of MS: A large multicenter study. *Mult Scler J* (2020) doi:10.1177/1352458519845844

8. Gaetani L, Di Carlo M, Brachelente G, Valletta F, Eusebi P, Mancini A, Gentili L, Borrelli A, Calabresi P, Sarchielli P, et al. Cerebrospinal fluid free light chains compared to oligoclonal bands as biomarkers in multiple sclerosis. *J Neuroimmunol* (2020) doi:10.1016/j.jneuroim.2019.577108

9. Sanz Diaz CT, de las Heras Flórez S, Carretero Perez M, Hernández Pérez MÁ, Martín García V. Evaluation of Kappa Index as a Tool in the Diagnosis of Multiple Sclerosis: Implementation in Routine Screening Procedure. *Front Neurol* (2021) doi:10.3389/fneur.2021.676527

10. Menéndez-Valladares P, García-Sánchez MI, Adorna Martínez M, García De Veas Silva JL, Bermudo Guitarte C, Izquierdo Ayuso G. Validation and meta-analysis of kappa index biomarker in multiple sclerosis diagnosis. *Autoimmun Rev* (2019) doi:10.1016/j.autrev.2018.07.010
